# Anxiety and depression in women cycling naturally and taking oral contraceptives – a daily diary study

**DOI:** 10.1192/j.eurpsy.2025.633

**Published:** 2025-08-26

**Authors:** M. Kowalczyk, M. Kornacka, I. Krejtz

**Affiliations:** 1 Psychology, SWPS University, Warsaw; 2 Psychology, SWPS University, Katowice, Poland

## Abstract

**Introduction:**

While the literature is inconsistent about the link between anxiety and oral contraceptives (OC) (Beltz FIN 2022; 67) the link between depression and OC is recognized in women who started taking OC during adolescence (Skovlund *et al.* JAMA Psychiatry 2016; 73). The review by Beltz gives many indications on how to conduct studies on these topics in a more reliable manner as there are a lot of variables to consider, such as assessing the exact phases of the menstrual cycle, comparing the active and inactive phases of OC or considering the types of OC. Other limitations described in the review are a lack of longitudinal studies and small sample sizes. The present study tried to take into account as many of these variables as possible.

**Objectives:**

Our study aimed to understand the differences between women taking OC and naturally cycling (NC) women regarding their daily levels of anxiety, depression, protective factors (self-esteem and daily satisfaction with life), perseverative cognition (worry and perseverative thinking) and stress.

**Methods:**

89 women (48 – OC; 41 – NC) participated in a daily diary study in which they completed an online diary for 15 days divided into 3 phases of the menstrual cycle: menstruation, follicular and luteal. The women using OC were further classified according to the androgenicity of their OC: androgenic progestins are derived from testosterone while anti-androgenic progestins block androgen receptors (Raudrant & Rabe Drugs 2003; 63).

**Results:**

There were no differences between groups in daily levels of anxiety, perseverative cognition, and stress. However, anti-androgenic OC users had higher levels of daily depression than NC women in all the phases of the menstrual cycle while androgenic OC users had higher levels of depression than NC women in the menses and follicular phases. Both groups of women taking OC had lower levels of daily self-esteem than NC women in all phases of the menstrual cycle. Androgenic OC users had higher daily satisfaction with life than anti-androgenic OC users in two phases.

**Conclusions:**

Our study found differences between groups in daily levels of depression and protective factors linked with it, namely self-esteem and satisfaction with life. Overall, OC users had higher levels of depression and lower levels of self-esteem than NC women. However androgenic OC users had higher daily satisfaction with life than anti-androgenic OC users. We believe that our study has contributed to the literature by conducting a longitudinal assessment of anxiety and depression on a daily level by comparing NC women and OC users during multiple phases of the menstrual cycle and taking into account the differences between androgenic and anti-androgenic OC. Future studies could explore the link between depression and its protective factors in women taking OC.

**Disclosure of Interest:**

None Declared

